# In Situ Gel Formation in Microporated Skin for Enhanced Topical Delivery of Niacinamide

**DOI:** 10.3390/pharmaceutics12050472

**Published:** 2020-05-21

**Authors:** Sonalika Bhattaccharjee, Moritz Beck-Broichsitter, Ajay K. Banga

**Affiliations:** 1 Center for Drug Delivery and Research, Department of Pharmaceutical Sciences, College of Pharmacy, Mercer University, Atlanta, GA 30341, USA; sonalika.arup.bhattaccharjee@live.mercer.edu; 2 MilliporeSigma a Business of Merck KGaA, Frankfurter Strasse 250, 64293 Darmstadt, Germany; moritz.beck-broichsitter@merckgroup.com

**Keywords:** Microneedle, Poly(lactide-*co*-glycolide), In situ gel, In vitro permeation testing, Topical drug delivery

## Abstract

Although used widely in cosmetic formulations, topical delivery of niacinamide (LogP = −0.35) is unfavorable by conventional means. Poly(lactide-*co*-glycolide) (PLGA) formulations, can undergo a sol-gel transition triggered by solvent exchange, entrapping molecules and sustaining their release. The current study aims to exploit the ability of PLGA to gel in situ and enhance the topical delivery of niacinamide in microporated skin. In vitro drug permeation studies were performed using vertical Franz diffusion cells. Microporation was performed using Dr. Pen^TM^ Ultima A6, where pre-treatment with a 1 mm needle-length for 10 s and a 0.5 mm needle-length for 5 s, both at 13,000 insertions/min were compared. The effect of different grades of PLGA, EXPANSORB^®^ DLG 50-2A (“low” molecular weight), and EXPANSORB^®^ DLG 50-8A (“high” molecular weight) on topical delivery was also determined. Formulations containing PLGA resulted in successful gelation in situ on application over microporated skin. A significantly higher amount of drug was found in the skin with the 0.5 mm treatment for 5 s (892 ± 36 µg/cm^2^) than with 1 mm for 10 s (167 ± 16 µg/cm^2^). Hence, the different grades of PLGA were evaluated with 0.5 mm, 5 s treatment, and a significantly larger amount was seen in skin with the higher rather than the lower molecular weight polymer (172 ± 53 µg/cm^2^).

## 1. Introduction

Niacinamide, also known as nicotinamide, the amide form of vitamin B_3_ (niacin), is an active ingredient in several dermatological and cosmetic formulations, ranging in concentration from 2% to 5% [1,2]. Being a coenzyme that regulates the formation of two important biochemical cofactors, nicotinamide adenine dinucleotide (NAD) and nicotinamide adenine dinucleotide phosphate (NADP), niacinamide mediates hydrogen transfer in cells, helps them proliferate, and repairs cell damage by neutralizing and absorbing free radicals. Topical application of niacinamide results in the aforementioned activity in skin cells, which forms the basis of the dermatological benefits associated with it [3,4,5]. Additionally, niacinamide increases protein synthesis and the production of ceramide and fatty acids within the skin. This results in the stabilization of epidermal barrier function, reduction in transepidermal water loss, and an increase in the moisture content in skin [6,7,8]. A combination of these effects contributes to smoother texture and appearance in aging skin, reduction of fine lines and wrinkles, and prevention of skin dehydration and sagging. This makes niacinamide the active ingredient of choice in more than 600 dermatological products [3,9,10,11], ranging across 45 different categories, including shampoos, skin moisturizers, and cleansing formulations.

Niacinamide’s anti-inflammatory effects also help prevent acne flare-ups and have been beneficial for treating rosacea and psoriasis [3,4]. Topical formulations containing 4% niacinamide have been known to treat moderate acne as effectively as 1% clindamycin, the drug of choice for acne treatment [12,13], and 2% topical niacinamide has been proven effective in inhibiting the production of facial sebum, adding synergistically to its anti-acne effects [14]. In 2005, the Cosmetic Ingredient Review Panel (CIRP) assessed the safety of niacinamide in more than 60 cosmetic formulations and deemed it safe for general use in cosmetics [15], making it suitable even for sensitive skin [1,4].

Although several therapeutic benefits have been attributed to this essential water-soluble vitamin, topical delivery of niacinamide, having a "negative" log partition coefficient of −0.35, is unfavorable by conventional means [2,16]. The stratum corneum, also known as the horny layer, is the uppermost layer of skin, consisting of corneocytes embedded in a lipid-enriched matrix at a thickness of approximately 10–15 μm. It forms a barrier that prevents the transport of hydrophilic molecules, such as niacinamide, into and across skin [2,17]. Recently, several microneedle (MN) based technologies have been emerging as means to disrupt the barrier properties of the stratum corneum, thus, enabling enhanced topical and transdermal delivery of therapeutic and cosmeceutical actives [17,18,19,20,21,22]. MNs are easy to administer and provide a patient-friendly alternative to deliver actives into and across the skin at a low cost [17]. By optimizing MN geometry, coupled with formulation strategies, controlled distribution of actives within the different layers of skin can be achieved. MNs puncture the stratum corneum without coming in contact with the underlying nerve fibers and blood vessels, present primarily in the dermis, resulting in a pain-free and minimally invasive means of delivering both small and large molecular weight, hydrophilic as well as lipophilic actives, without side effects at the application site [17,23]. Several MN devices have already obtained FDA clearance, primarily for cosmetic applications, such as DermaRoller^®^ (DermaRoller^®^ GmbH, Wolfenbüttel, Germany), Dermapen^®^ (EquipMED^™^, Belrose, Australia) and Revive^™^ HAP (AMIEA MED, Berlin, Germany) [17,24]. MNs are frequently employed in cosmetic clinics for several conditions like scar reduction post-acne, burn treatment, cellulite reduction, hair restoration, hyperpigmentation, and melasma recovery [24,25,26]. MNs cause percutaneous collagen induction for dermal repair and are also applied as a pre-treatment to enhance the penetration of topically applied cosmeceutical actives [24,25,26,27,28,29]. Recently, the Dr. Pen^™^ Ultima A6 (Dr. Pen Inc., San Jose, CA, USA) device was introduced for general cosmetic usage as an automated MN system, with a streamlined wireless design that can be used at home, without professional training or supervision [30].

In the current study, an in situ gelling formulation was developed for application on microporated skin, for the slow topical delivery of niacinamide. Pre-treatment of skin using Dr. Pen^™^ Ultima A6 prior to the application of niacinamide was thought to be beneficial for not only disrupting stratum corneum, the barrier to the transport of hydrophilic niacinamide into skin, but also for having a synergistic effect on the treatment of several dermatological conditions to be targeted by this novel approach [31]. However, to ensure maximum topical delivery of niacinamide and prevent its loss to systemic circulation, an in situ gelling formulation was designed to gel within the microchannels created in skin and sustain the release of the active into the layers of skin. To impart the ability to gel in situ, poly(lactide-*co*-glycolide) (PLGA), a biodegradable and biocompatible co-polymer used extensively in drug delivery systems and devices, was incorporated in the formulation [32,33]. PLGA can undergo a sol-gel transition triggered by solvent exchange, entrapping drugs in a polymeric matrix, and sustaining their release, a property that has been frequently exploited to prepare in situ-forming implants [34,35] and micro/nano-particles [34,36,37,38]. However, the aim of this study was to develop a novel approach to enhance the topical delivery of a hydrophilic active, niacinamide, by forming an in situ gel within microchannels created in skin.

## 2. Materials and Methods

### 2.1. Materials

Niacinamide and polyethylene glycol 400 (PEG 400) were purchased from Sigma Aldrich (St Louis, MO, USA). PLGA polymers, EXPANSORB^®^ DLG 50-2A (1:1 lactide/glycolide ratio; molecular weight (MW) range: 15–30 kDa) and EXPANSORB^®^ DLG 50-8A (1:1 lactide/glycolide ratio; MW range: 80–130 kDa) were kindly donated by Merck KGaA (Darmstadt, Germany). Dr. Pen™ Ultima A6 (MN device) was purchased from Dr. Pen Inc. (San Jose, CA, USA). Porcine ear skin was obtained from a local slaughterhouse (Atlanta, GA, USA). Dimethyl sulfoxide (DMSO, Procipient^®^) was obtained as a sample from Gaylord Chemical (Slidell, LA, USA). Fluoresoft^®^ was purchased from Alden Optical (Lancaster, NY, USA). Potassium phosphate dibasic, phosphate-buffered saline 10× solution, HPLC grade acetonitrile, and orthophosphoric acid were purchased from Fisher Scientific (Bridgewater, NJ, USA). HPLC grade methanol was obtained from Medsupply Partners (Atlanta, GA, USA).

### 2.2. Solubility Studies

The solubility of niacinamide was determined in DMSO, PEG 400, and pH 7.4 phosphate-buffered saline (PBS, containing 11.9 mM of phosphates, 137 mM of sodium chloride and 2.7 mM potassium chloride). After overnight shaking, the resultant mixture of niacinamide in each solvent was centrifuged at 15,000 rpm at 25 °C for 30 min. The saturated solvent layer, if applicable, was appropriately diluted and analyzed for saturation solubility using the HPLC-UV method described in Section 2.5. The saturation solubility in each solvent was assessed in triplicate.

### 2.3. Preparation of In Situ Formulation

In situ gelling formulations were prepared with PLGA as the in situ gelling polymer dissolved in DMSO as the vehicle and PEG 400 as a plasticizer (Table 1). Control formulation without PLGA was also prepared. As listed in Table 1, PLGA was dissolved in DMSO and PEG 400 for 72 h on a slow rotary mixer with intermittent vigorous shaking on a vortex shaker. A homogenous blend of the components was prepared by weight before the addition of niacinamide (4% w/w in the blank blend). The concentration of each component in the formulations prepared was lower than the maximum concentration listed in the inactive ingredient database of approved drug products [39]. The color and appearance of the formulations were examined visually over three months. Varying concentrations of the components were examined to determine the ratio of polymer and solvents to obtain a spreadable solution.

### 2.4. In Vitro Permeation Testing

#### 2.4.1. Preparation of Skin

After washing the freshly excised porcine ears, full-thickness skin was manually removed from the cartilage using a scalpel and scissors. The separated full-thickness porcine ear skin was stored at −80 °C until further use. Prior to in vitro permeation testing, the stored skin was thawed at room temperature with PBS solution, trimmed, and the layers of fat underneath were removed manually using forceps and scissors. The skin was cut to an appropriate size of 2 cm × 2 cm, and the thickness was measured using a thickness gauge (Cedarhurst, NY, USA).

#### 2.4.2. Measurement of Skin Electrical Resistance

The barrier integrity of the skin pieces was evaluated by measuring the electrical resistance offered by them. Silver-silver chloride electrodes attached to an arbitrary waveform generator (Agilent 33220A, 20 MHz Function) and 34410A 6 ½ digital multimeter (Agilent Technologies, CA, USA) were used for measurement. Skin pieces were mounted on vertical Franz diffusion cells and allowed to equilibrate for 30 min after the addition of 300 μL and 5 mL of PBS in the donor and receptor, respectively. After equilibration, the silver wire and the silver chloride electrodes were placed in the receptor and donor compartment, respectively. A load resistor (R_L_) was attached in series with skin, and the drop in voltage across the entire circuit (V_O_) and skin (V_S_), as displayed on the multimeter, was recorded. Skin resistance (R_S_) was calculated using equation 1:

(1) $$R_{S}=\frac{V_{S}R_{L}}{\left(V_{O}-V_{S}\right)}$$

where, V_O_ and R_L_ were 100 mV and 100 kΩ, respectively. Skin pieces with resistance greater than 10 kΩ were selected for the permeation study.

#### 2.4.3. Microneedle Treatment of Skin

The freshly defatted porcine ear skin pieces were placed flat on four layers of parafilm (Parafilm M Laboratory film; Neenah, WI, USA) with the stratum corneum side up. For groups pre-treated with maltose MNs, manual insertion using an MN applicator was performed for 2 min. In the case of groups treated with Dr. Pen^™^ Ultima A6, the MN cartridge was fixed onto the automated device and then adjusted to the required needle length. The device, along with the affixed cartilage, was held vertically on the skin piece, and motorized stamping was performed at the lowest speed setting (13,000 insertions/min), controlled by the start/stop button. MN length and skin resistance, as described in Section 2.4.2, was evaluated after treatment to confirm successful microporation. Dr. Pen^™^ Ultima A6 cartridges were visualized under a polarized light microscope (Leica DM750) to evaluate MN length and geometry. Images were taken using a Leica DFC camera attached to the microscope (Leica Microsystems Inc., Buffalo Grove, IL, USA).

##### Dye Binding Studies

Methylene blue solution (1% w/v in deionized water) was used to visualize the surface of MN-treated skin. The solution was applied immediately after treatment and swabbed off using Kimwipes (Kimberly-Clark Worldwide Inc., Irving, TX, USA) and alcohol swabs (Alcohol Prep, Curity^TM^, Covidien, Walpole, MA, USA). The stained area was visualized using ProScopeHR Digital USB Microscope (Bodelin Technologies, Oregon City, OR, USA), and the surface area of the stained regions was calculated using ImageJ software (National Institutes of Health, Bethesda, MD, USA).

##### Confocal Microscopy

The depth of microchannels created by the MN device was measured using confocal microscopy. Freshly defatted porcine ear skin was treated with the device as described above. Two treatment groups were tested: (a) 0.5 mm needle length applied for 5 s, and (b) 1 mm needle length applied for 10 s, both at 13,000 insertions/min. Fluoresoft^®^ (0.35%, 200 μL) was applied on the microchannels, and after a min, the excessive calcein was removed using Kimwipes and alcohol swabs. The treated skin was placed on a microscope slide and scanned using a computerized Leica SP8 confocal laser microscope (Leica Microsystems GmbH, Wetzlar, Germany) with a 10× objective at an excitation wavelength of 496 nm. Resultant images were processed by the Leica Application Suite-Advanced Fluorescence (LAS-AF) software (Leica Microsystems Inc., Buffalo Grove, IL, USA). X-Z sectioning was employed to study the distribution pattern of calcein in the channels and the depth of the created microchannels with a step size of 10 μm.

#### 2.4.4. Histological Evaluation

Full-thickness porcine ear skin was treated with 0.5 mm needle length for 5 s using the MN device, followed by the application of formulation with EXPANSORB^®^ DLG 50-8A (F2; Table 1). Methylene blue solution (1% w/v in deionized water) was used to stain the formulation followed by hematoxylin and eosin staining. Vertical sections (10 μm-thick) of MN-treated skin, with and without in situ formed gel, before and after hematoxylin and eosin staining was visualized under a Leica DM 750 optical microscope (Leica Microsystems Inc., Buffalo Grove, IL, USA).

#### 2.4.5. Evaluation of Topical Delivery of Niacinamide

In vitro permeation studies (*n* ≥ 4) were carried out on full-thickness porcine ear skin using static diffusion Franz cells. The receptor compartment was maintained at 37 °C by a circulating water jacket, and sink condition was maintained using PBS (5.0 mL). Skin was mounted on the surface of the receptor compartment with an orifice of 0.64 cm^2^ and clamped in between the donor and receptor compartments. Formulation (200 μL) was applied using a positive displacement pipette (Eppendorf Repeater^®^ E3X, Eppendorf North America, Hauppauge, NY, USA) on the exposed permeation area, with or without pre-treatment with MNs (described in Section 2.4.3; Table 2). Samples of receptor solution (300 μL) were collected at predetermined time points (0, 1, 2, 4, 6, 8, 24, 48, and 72 h) and replaced with an equal volume of fresh PBS. The skin temperature was measured using an infrared thermometer and was maintained at 30–33 °C for the duration of the study.

At the end of the permeation study, skin was wiped with Kimwipes to remove residual formulation, followed by removal of unabsorbed formulation using lauryl ether sulfate and DI water. Next, the epidermis (i.e., the stratum corneum and viable epidermis) from the permeation area of the skin was separated using forceps and minced manually using surgical scissors, extracted for niacinamide with methanol, filtered, and analyzed using HPLC. The amount of niacinamide remaining in the dermis was extracted and quantified by the same method.

### 2.5. Analytical Method

The quantification of niacinamide was achieved using a Waters Alliance 2695 separation module (Milford, MA, USA) coupled with a Waters 996 photodiode array detector. The separation was carried out on a Phenomenex Kinetex^®^ C18 column (5 μm, 250 mm × 4.6 mm) maintained at 35 °C. An isocratic profile was applied with acetonitrile and potassium phosphate dibasic buffer (50 mM, pH 7.0) at a ratio of 5:95 (v/v). The elution was performed at the flow rate of 1 mL/min with a run time of 10 min. For all aqueous samples, 20 μL was injected into the system, and 5 μL was injected for the methanol samples. Peak area values were collected at a wavelength of 262 nm for niacinamide [40]. This method was validated regarding linearity, dynamic range, sensitivity (LOD and LOQ), within-batch (n = 3), and inter-batch (n = 3) accuracy and precision [41].

### 2.6. Data Analysis/Statistics

All results have been reported as the mean with standard error (SE). Statistical calculations were performed using GraphPad Prism7 (GraphPad Software, San Diego, CA). The Kruskal–Wallis test was used to compare the results of different groups, and a statistically significant difference was depicted by a *p* value of < 0.05.

## 3. Results

### 3.1. Solubility Study

Niacinamide was found to be very soluble in DMSO (395 ± 11 mg/mL) and PEG 400 (362 ± 5.96 mg/mL), indicated by no precipitation in the saturated supernatant post-centrifugation. The saturation solubility in PBS was measured to be 114 ± 2 mg/mL.

### 3.2. Preparation of In Situ Formulation

The formulations prepared (Table 1) were found to be visually homogenous and clear, with no phase separation over 3 months. Formulations F1 and F2 had a slight yellow coloration and were found to be viscous, while the control formulation was a colorless fluid. F2, the formulation containing a higher molecular weight of PLGA (EXPANSORB^®^ DLG 50-8A), demonstrated a higher viscosity in comparison to the lower molecular weight PLGA (EXPANSORB^®^ DLG 50-2A) formulation, F1.

### 3.3. Microneedle Treatment

**Figure 1:**
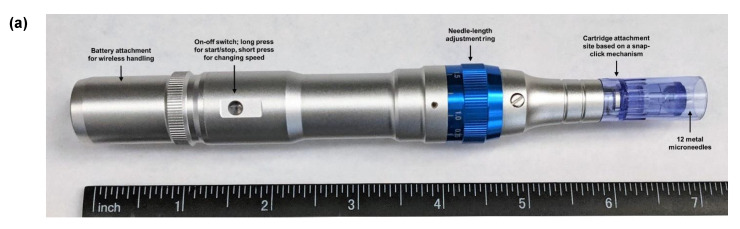

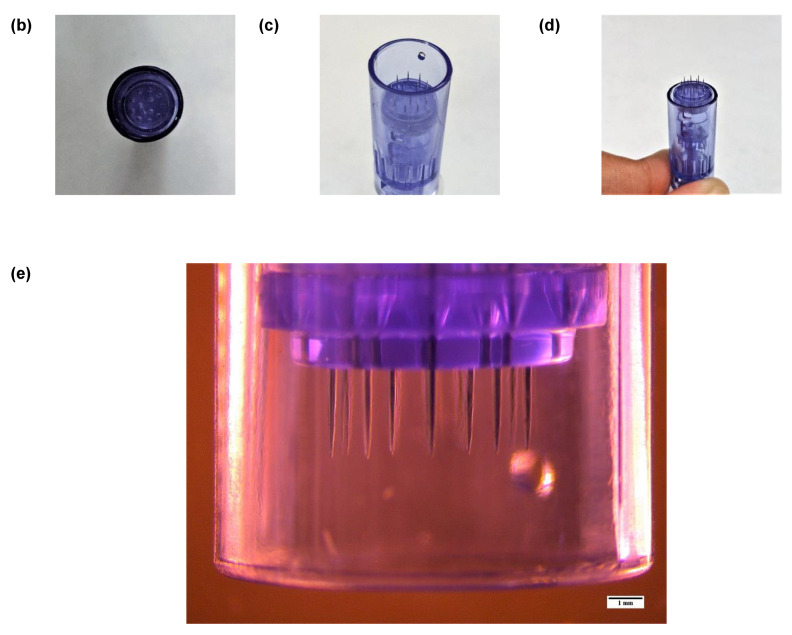
Image of Dr. Pen™ Ultima A6 with (**a**) description of the various parts of the device, (**b**) a top view of the cartridge displaying the arrangement of 12 microneedles and (**c**) a side view of the cartridge with the needles in the "off" position and (**d**) the needles pushed out of the cartridge at its maximum length. Image of (**e**) the cartridge visualized under a polarized light microscope. Scale bar represents 1 mm.

Images of Dr. Pen^TM^ Ultima A6, the MN device, are presented in Figure 1 with a microscopic evaluation of the needles in the cartridge used for in vitro permeation. The needles were found to be long, thin and cylindrical, tapering to a point at the exposed end.

MN treatment with the device was found to be successful with a significant reduction in skin electrical resistance after treatment. Needle length of 1 mm, applied for 10 s resulted in 92 ± 1% reduction, while 0.5 mm needle length, applied for 5 s reduced skin resistance by 89 ± 1%.

**Figure 2:**
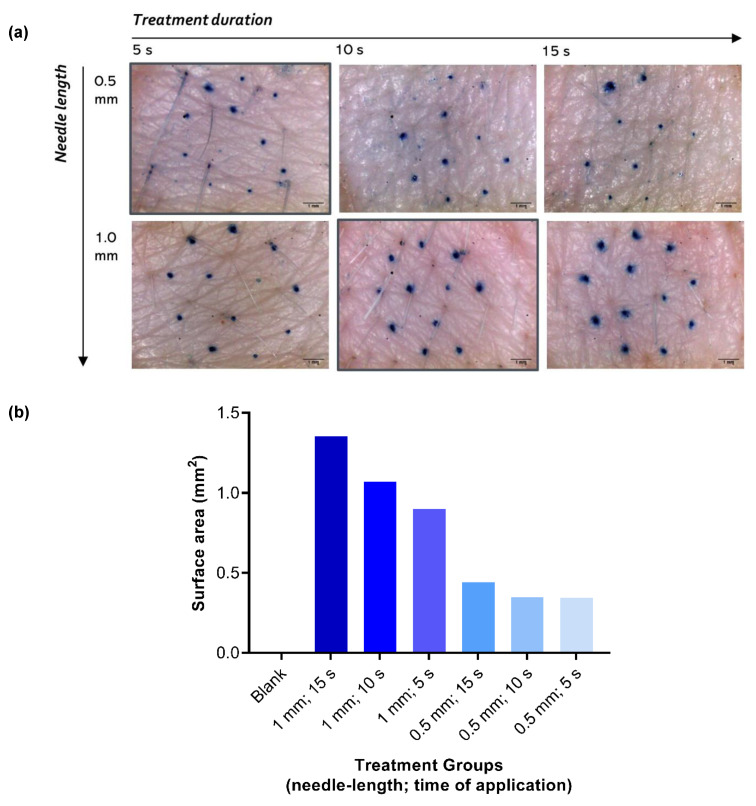
(**a**) Visualization of microchannels created using Dr. Pen^TM^ Ultima A6 at 13,000 insertions/min, stained using methylene blue (1% w/v in deionized water) and observed using ProScope HR Digital USB Microscope. (**b**) The surface area of the pores was calculated using ImageJ software (*n* = 1). Scale bar represents 1 mm.

Dye-binding studies enabled the visualization of the hydrophilic pores created by the MN device (Figure 2). Needle lengths lower than 0.5 mm, applied for 5 s, resulted in incomplete microporation (<10 microchannels formed by the 12 needles in a cartridge). Longer than 10 s treatment with 1 mm needle length resulted in excessive diffusion of dye from the pores formed. The surface area of the pores formed ranged from 0.345 mm^2^ (0.5 mm needle length; 5 s) to 1.354 mm^2^ (1 mm needle length; 15 s), as compared in Figure 2b.

**Figure 3:**
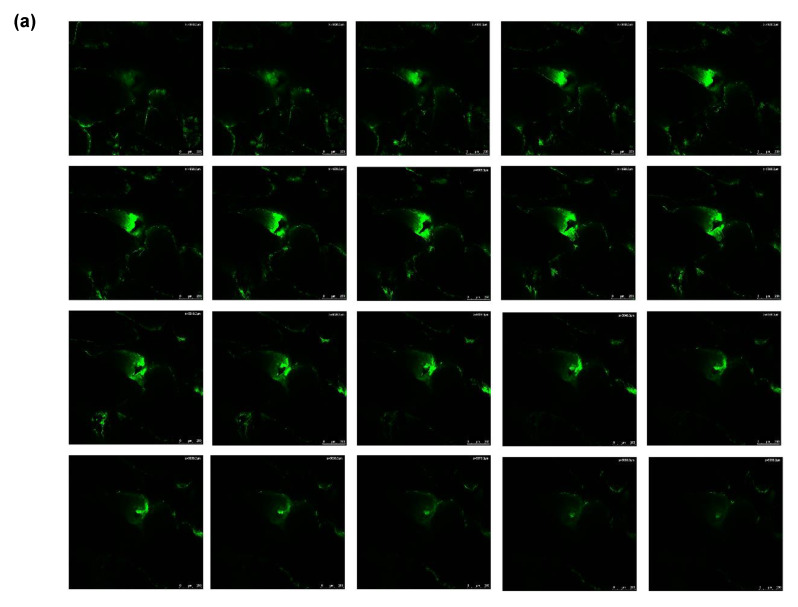

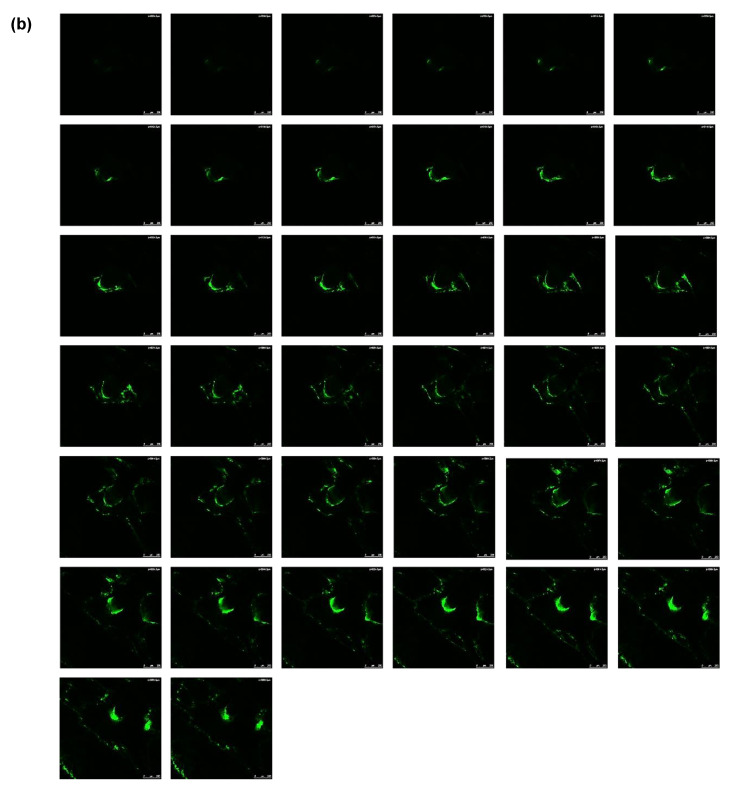
Confocal microscopy z-stack of microchannels created by Dr. Pen^TM^ Ultima A6 with (**a**) 0.5 mm needle length applied for 5 s, and (**b**) 1 mm needle length applied for 10 s, both at 13,000 insertions/min. Scale bar represents 250 µm.

The depth and surface area of the microchannels created were studied by Leica SP8 confocal laser microscopy without any dimensional distortions during physical sectioning. Hair follicles were not observed in the confocal images (Figure 3), confirming the diffusion of calcein solely through the microchannels created, and not the follicular pathway. Deeper microchannels were formed with a 1 mm treatment for 10 s (380 µm) as compared to 0.5 mm for 5 s (200 µm). The morphology of the micropores followed a conical pattern for both treatments. However, irregularities in the conical pattern and greater diffusion of calcein dye, staining only the inner walls of the microchannels, was observed on treatment with 1 mm needle length treatment for 10 s.

### 3.4. Histological Evaluation

**Figure 4:**
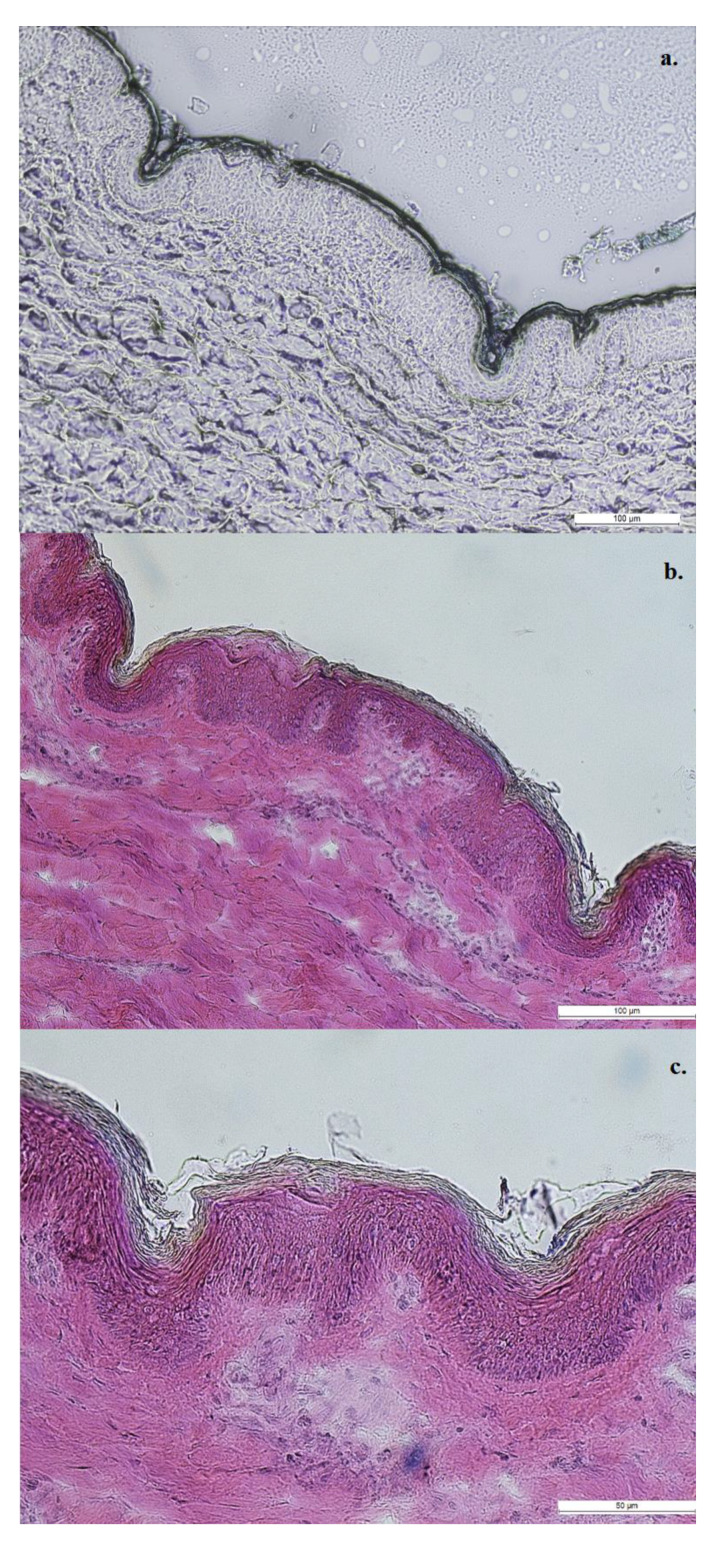
Histological evaluation of (**a**) the formation of gel in situ in successfully microporated skin (methylene blue solution used to stain the formulation) followed by (**b**,**c**) hematoxylin and eosin staining. Scale bar represents (**a**,**b**) 100 µm and (**c**) 50 µm.

Successful microporation of skin and disruption of the stratum corneum was seen with MN-pretreatment (Figure 4). The skin portions around the microchannel maintained typical structure with intact stratum corneum, whereas the microchannels were deep indentations with disrupted stratum corneum at the base, extending through the epidermis. As multiple insertions occur at the same site (13,000 insertions/min), the disrupted stratum corneum was observed to have deposited in the microchannels created. In addition, localization of blue-stained tissue could be observed at the base of microchannels in Figure 4a, along with the stained in situ gel forming a depot in the created microchannels.

#### 3.4.1. Topical Delivery of Niacinamide with Maltose MNs

**Figure 5:**
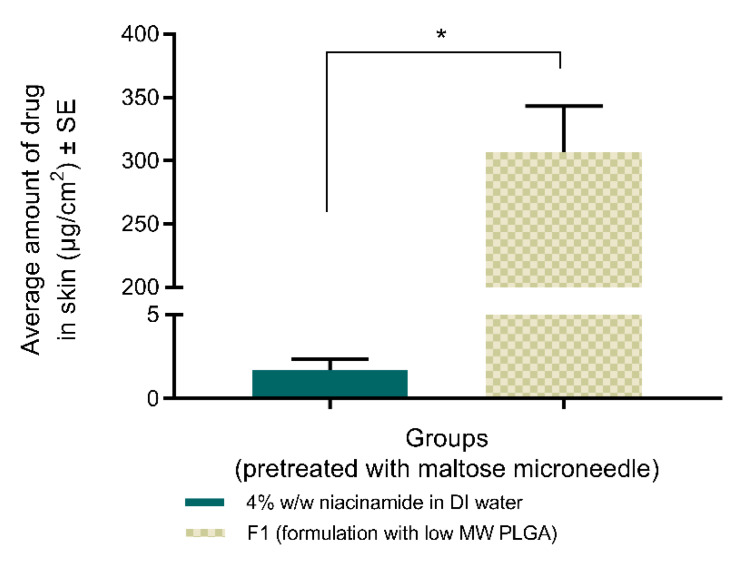
Average amount of niacinamide delivered to porcine ear skin (epidermis and dermis) after 72 h. Groups were treated with maltose microneedles prior to application of formulations. ‘F1’ denotes 4% w/w niacinamide in a solution of PLGA (EXPANSORB^®^ DLG 50-2A), DMSO, and PEG 400 (2:1:7 w/w) to form the in situ gel. (Kruskal–Wallis test: *, *p* ≤ 0.05; *n* = 4).

For skin treated with maltose MN, at the end of the study, formulation F1 formed a semi-solid film over the permeation area while drug solution in DI water remained as a liquid. Additionally, F1 retained a significantly higher amount of drug in skin (307 ± 36 µg/cm^2^), compared to the aqueous solution (1.7 ± 0.6 µg/cm^2^), as shown in Figure 5. On skin with no treatment, 4% w/w solution of niacinamide in DI water delivered a significantly higher amount in skin (50 ± 19 µg/cm^2^) compared to the same solution applied on maltose MN-treated skin, but was significantly lower than the delivery with F1 on maltose MN-treated skin.

#### 3.4.2. Topical Delivery of Niacinamide with MN Device

**Figure 6:**
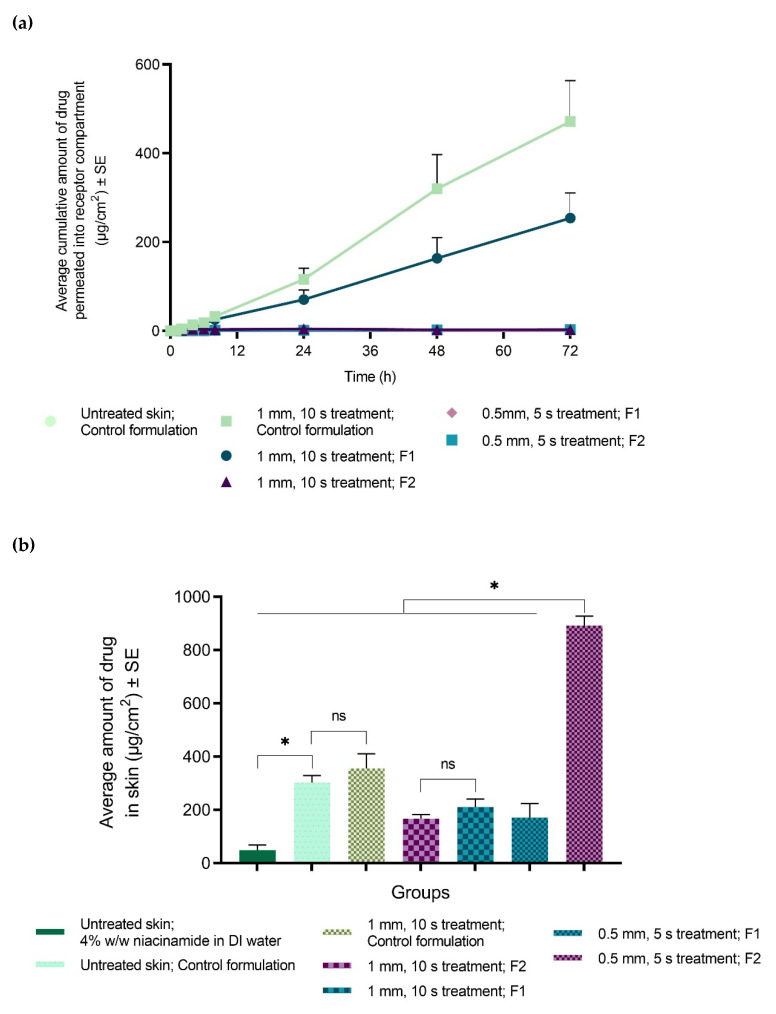
Average amount of niacinamide delivered (**a**) across porcine ear skin (receptor compartment and (**b**) into the skin (epidermis and dermis) after 72 h. Groups were either treated with Dr. Pen^™^ Ultima A6 (1 mm needle length for 10 s or 0.5 mm needle length for 5 s, at 13,000 insertions/min) prior to the application of formulations or applied on intreated skin, as specified. ‘F1’ denotes 4% w/w niacinamide in an in situ gel formulation containing PLGA (EXPANSORB^®^ DLG 50-2A; “low” MW) and ‘F2’ denotes a similar formulation with PLGA (EXPANSORB^®^ DLG 50-8A; “high” MW). Control formulation does not contain PLGA. (Kruskal–Wallis test: *, *p* ≤ 0.05; *n* = 4).

Successful gelation in skin treated with the MN device was observed with PLGA-containing formulations, F1, and F2. Although the incorporation of PLGA reduced the amount of niacinamide in the receptor compartment and sustained the release across skin (Figure 6a), studies conducted with 1 mm, 10 s setting did not show statistically different amounts of niacinamide in skin between the different grades of PLGA (167 ± 16 µg/cm^2^ with F2; 211 ± 30 µg/cm^2^ with F1). Significantly higher topical delivery was seen with the control formulation (356 ± 55 µg/cm^2^, Figure 6b) in comparison to the PLGA-containing formulation F2. However, a significantly higher amount of drug was found in skin with 0.5 mm treatment for 5 s (892 ± 36 µg/cm^2^), than with 1 mm for 10 s (167 ± 16 µg/cm^2^), when tested with F2 (formulation containing higher molecular weight PLGA, EXPANSORB^®^ DLG 50-8A). The different grades of PLGA were compared with 0.5mm, 5 s pre-treatment and significantly higher amount was seen in skin with formulation F2 (892 ± 36 µg/cm^2^, containing EXPANSORB^®^ DLG 50-8A) compared to F1 (172 ± 53 µg/cm^2^, containing EXPANSORB^®^ DLG 50-2A). Additionally, a significantly higher amount of niacinamide was delivered topically with the control formulation (303 ± 27 µg/cm^2^) on untreated skin when compared to 4% w/w solution of niacinamide in DI water (50 ± 19 µg/cm^2^). However, application of the control formulation on intact skin (303 ± 27 µg/cm^2^) in comparison to MN-treated skin (1 mm, 10 s; 356 ± 55 µg/cm^2^) did not show significant difference in dermal delivery, further confirming that microporation of skin by itself was not responsible for enhanced dermal delivery, but necessary to ensure gelation of the PLGA-containing formulations in situ.

### 3.5. Analytical Method

The HPLC method was validated successfully for linearity, dynamic range, sensitivity (LOD and LOQ), within-batch and inter-batch accuracy, and precision. The LOD and LOQ were 0.17 and 0.52 µg/ml, respectively. The calibration curves showed linearity across the tested range of 0.1–50 µg/ml with r-square >0.9999 [40]. Within-batch and inter-batch accuracy and precision lay within the acceptable range.

## 4. Discussion

Several applications have been attributed to in situ forming drug delivery systems [34,38,42,43,44] as they can provide sustained release of drugs, reduce the frequency of application, ease administration and improve patient compliance and comfort [45]. In situ forming systems can adapt one of the following mechanisms to transition from sol-to-gel: (i) solidification of hydrogels/organogels; (ii) cross-linking and (iii) precipitation [34], which can be triggered by different phenomena, including solvent exchange and changes in the pH or temperature [46]. In our study, in situ gelation was adapted as a novel approach to sustain topical delivery of niacinamide in MN-treated skin.

Biodegradable polymers have been investigated for controlled drug delivery since their introduction as bioresorbable devices about four decades ago [32]. Recently, Khan et al. explored sustained transdermal delivery by MN-assisted in situ gels for the first time, in which change in temperature within microchannels created in skin was used to induce sol-gel transition of poloxamer. Poloxamer undergoes a reverse thermal gelation phenomenon, where a formulation containing it remains fluid below its characteristic sol-gel transition temperature and above it, turns semi-solid [47]. However, its application in drug delivery systems is limited by its long-term instability, poor mechanical properties, and short residence times owing to rapid dissolution in vivo [48,49,50]. By contrast, PLGA is an attractive polymeric candidate used to fabricate devices for drug delivery. It belongs to a family of FDA-approved biodegradable polymers that are mechanically strong, highly biocompatible, and investigated extensively for various applications and drug types. Additionally, PLGA offers high tunability of release kinetics by controlling the polymer molecular weight, the ratio of lactide to glycolide, and drug concentration to achieve a desired dosage and release interval [32]. In our study, solvent-exchange to induce in situ gelation of a PLGA formulation was exploited for the sustained delivery of niacinamide.

PLGA, being a water-insoluble polymer, when dissolved in an organic solvent, precipitates when exposed to an aqueous environment. Phase separation occurs in the aqueous medium as the dissolving solvent diffuses towards the surrounding aqueous environment while at the same time, water/body fluids from the surroundings penetrate the organic phase. This results in a depot entrapping the drug at the application site [34].

Drug release from a PLGA depot is generally characterized by an initial burst during the solidification of the matrix, followed by diffusion and, finally, drug release driven by degradation and erosion [34]. Initial burst release can be reduced by modifying the type of solvent, the lactide to glycolide ratio of the polymer, the concentration and MW (viscosity) of PLGA, and incorporation of plasticizer or surfactant [51]. Although *N*-methyl-2-pyrrolidone is a frequently used water-miscible solvent for PLGA [34], the current study involved the use of DMSO, as a significantly reduced burst release has been reported by Lin et al. with DMSO as the solvent, compared to *N*-methyl-2-pyrrolidone and ethanol [52]. Lin et al. investigated the effect of DMSO, NMP, and ethanol on the release kinetics of risperidone from a PLGA-based depot. The reason for the lower initial burst release with DMSO was attributed to two factors: (i) DMSO demonstrated a lower solvent release rate, resulting in a slower mass transfer rate (drug diffusion rate) during solvent exchange and hence, a lower burst release; (ii) DMSO increased the viscosity and intermolecular force of the depot systems, which further decreased the diffusion rate of DMSO, resulting in less water required to initiate the precipitation of PLGA. This led to superficial solidification and encapsulation of the solvent and drug in the form of a skin (shell) at the interface of solvent exchange. The shell around the system was found to be an obstacle for the free drug to be "released" in a burst. Higher concentration or molecular weight of PLGA has a similar effect in reducing the initial burst of drug [52,53]. Additionally, a lactide to glycolide ratio of 50:50 has the fastest degradation rate, lowers burst release, and is, therefore, one of the most frequently used PLGA polymer types [54], which was also employed in our studies. A higher percentage of lactic acid imparts greater hydrophobicity to the polymer, and consequently, less water is absorbed, and the degradation of PLGA is slower [51,54]. Finally, the incorporation of PEG 400 has been reported to decrease the drug initial burst, possibly by its plasticizing effect on a PLGA matrix system. The solubilizing power of PEG 400 allows uniform distribution of drug particles inside the PLGA matrix, preventing adsorption at the surface, and hence, lower burst effect [51]. Thus, the solubility of niacinamide was investigated in DMSO and PEG 400 to ensure complete dissolution of drug at 4% drug loading, and the in situ gel formulation was optimized to F1 and F2, as described in Table 1. Formulations were prepared with EXPANSORB^®^ DLG 50-2A (1:1 lactide/glycolide ratio; MW range: 15–30 kDa) and EXPANSORB^®^ DLG 50-8A (1:1 lactide/glycolide ratio; MW range: 80–130 kDa) to evaluate the effect of different grades of PLGA on topical delivery of niacinamide.

In our study, the aqueous environment for PLGA to gel within microchannels in skin was provided by interstitial fluid (ISF) released into these microchannels due to MN pre-treatment [55]. MNs are known to create micron-sized pathways for ISF to transport out of skin and reside in the microchannels created, until pore-closure [55]. 

Dr. Pen^TM^ Ultima A6 features different kinds of cartridges with a varying number of needles in an MN array, as well as different MN geometries suited for different dermatological applications. The device was found to be superior to dissolving MNs as it allows users to vary the depth of penetration in skin by adjusting the length of the needles that are exposed beyond the cartridge. Available needle length for penetration can be changed from 0.25 to 2.5 mm. Additionally, the device allows five speeds of application to choose from (13,000, 14,000, 15,000, 16,000, or 17,500 insertions/min), which varies the rate of motorized stamping of the MNs in an in-and-out motion and eliminates the need for an additional applicator. The MN device is also marketed for commercial usage at home. In our study, the anti-wrinkle cartridge containing 12 needles, as shown in Figure 1, was used. As the needles are prepared with stainless steel, they do not leave any residue in the microchannels formed. Additionally, it allows application over large surface areas throughout the body, and the area of application is not limited by the MN array size. Based on preliminary dye-binding studies performed with methylene blue (Figure 2), a needle length of 0.5 mm for 5 s and 1 mm needle-length applied for 10 s were selected. The lowest speed setting of 13,000 insertions/min was chosen to prevent excessive vibrations of the device during application on skin.

Although MN length directly affects microchannel robustness and geometry, the effect of pore closure on drug delivery with different MN pre-treatments must also be taken into consideration [56]. Pore closure can be further delayed by occlusion of microchannels created, as employed in our study. Occlusion in general delays barrier recovery [57,58,59,60], and in combination with MN technology, it can result in a prolonged therapeutic window. Previous studies in our lab have demonstrated a delayed pore-closure for up to 72 h in MN-treated skin on the application of a film or a solution [61]. Hence, considering that the pores created by the MN device exhibit a similar delay in pore-closure after application of the in situ forming gel, in vitro permeation evaluation was performed for 72 h in the current study.

In vitro permeation studies on porcine ear skin, demonstrated the success of the designed formulations on the enhanced topical delivery of niacinamide. Initial studies performed with maltose MNs established proof of concept of in situ gelation within microchannels, in addition to histological evaluation of the in situ gelation as observed in Figure 4. Although a significantly higher amount of drug in skin (307 ± 36 µg/cm^2^) was seen with F1-formulation, compared to the aqueous solution (1.7 ± 0.6 µg/cm^2^, Figure 5) on MN-treated skin, the retention of niacinamide in skin was lower with 4% w/w aqueous solution on MN-treated skin, compared to untreated (50 ± 19 µg/cm^2^). Thus, enhanced topical delivery on the application of F1 on microporated skin was attributed to the application of the in situ gel on microporated skin, and not due to microporation of skin alone. Although microporation of skin by itself did not enhance dermal delivery of niacinamide, it was necessary to facilitate gelation of the PLGA-containing formulations in situ.

Delivery by a formulation without PLGA (4% drug in 1:7 DMSO and PEG 400; control formulation, Table 1) was evaluated to investigate the effect of DMSO and PEG 400 on topical delivery of niacinamide. Although PLGA was found to be responsible for in situ gelling of the formulation, DMSO and PEG 400 by themselves increased the amount of niacinamide in untreated skin (303 ± 27 µg/cm^2^; control formulation) in comparison to the 4% w/w aqueous solution (50 ± 19 µg/cm^2^).

Studies conducted with a 1 mm needle length for 10 s did not show statistically different amounts of drug in skin between the different grades of PLGA (167 ± 16 µg/cm^2^ with F2; 211 ± 30 µg/cm^2^ with F1). However, a significantly higher amount of drug was found in skin with 0.5 mm treatment for 5 s (892 ± 36 µg/cm^2^), than with 1 mm for 10 s (167 ± 16 µg/cm^2^) when formulation F2, containing the higher molecular weight of PLGA was applied. Based on the results of confocal microscopy, deeper microchannels were formed with a 1 mm treatment for 10 s (380 µm) as compared to 5 mm for 5 s (200 µm) which could have resulted in higher systemic delivery of niacinamide with 1 mm, 10 s application, rather than retention of the drug in the skin. The microchannels formed by a 1 mm needle-length for 10 s reaches the dermis of skin and hence, was found to be too deep for topical delivery of niacinamide. Additionally, the distribution of calcein dye during confocal microscopy indicated rapid diffusion of the hydrophilic dye with 1 mm, 10 s treatment.

Hence, the effect of different grades of PLGA was reevaluated with 0.5 mm, 5 s treatment and a significantly higher amount was seen in skin with F2 (892 ± 36 µg/cm^2^) in comparison to F1 (172 ± 53 µg/cm^2^). As higher molecular weight PLGA, as used in F2, has a higher viscosity, it lowers initial burst release and slows diffusion of solvent and drug from the matrix [51,52]. Thus, drug loss to the receptor compartment was lower with F2 than with F1, and topical delivery of niacinamide was higher with F2.

## 5. Conclusions

Successful gelation of a PLGA-containing solution in situ with slow delivery of niacinamide was achieved. A treatment using 0.5 mm needle-length for 5 s with an MN device followed by the application of a formulation with EXPANSORB^®^ DLG 50-8A resulted in the highest delivery into skin.

## Figures and Tables

**Table 1:** Niacinamide formulations prepared with PLGA as the in situ gelling polymer.

Formulations Prepared ^1^	Components (% w/w)			
	EXPANSORB^®^ DLG 50-2A	EXPANSORB^®^ DLG 50-8A	DMSO	PEG 400
Control	-	-	12.50	87.50
F1 *(“Low” MW PLGA ^2^ formulation)*	20.00	-	10.00	70.00
F2 *(“High” MW PLGA ^3^ formulation)*	-	20.00	10.00	70.00

^1^ Niacinamide (4% w/w) was added to the components listed, after complete dissolution of PLGA. The ratio of DMSO to PEG 400 was maintained as 1:7 in all formulations; ^2^ “Low” MW PLGA used to prepare formulation: EXPANSORB^®^ DLG 50-2A (1:1 lactide/glycolide ratio; MW range: 15–30 kDa); ^3^ "High" MW PLGA used to prepare formulation: EXPANSORB^®^ DLG 50-8A (1:1 lactide/glycolide ratio; MW range: 80–130 kDa).

**Table 2:** Study design for in vitro permeation testing.

Treatment ^1^			Formulation Applied ^2^			
			Control	F1	F2	Solution in Deionized Water
No treatment			✓	✕	✕	✓
Dr. Pen^™^ Ultima A6	*1 mm needle length*	*10 s application*	✓	✓	✓	✕
	*0.5 mm needle length*	*5 s application*	✕	✓	✓	✕
Maltose microneedle	*0.5 mm needle length*	*2 min application*	✕	✓	✕	✓

^1^ Pre-treatment of porcine ear skin prior to application of formulation; ^2^ All formulations contain 4% w/w niacinamide.
